# Detecting non-coding selective pressure in coding regions

**DOI:** 10.1186/1471-2148-7-S1-S9

**Published:** 2007-02-08

**Authors:** Hui Chen, Mathieu Blanchette

**Affiliations:** 1 McGill Centre for Bioinformatics, McGill University, 3775 University St., room 332, Montreal, QC, Canada H3A 2B4

## Abstract

**Background:**

Comparative genomics approaches, where orthologous DNA regions are compared and inter-species conserved regions are identified, have proven extremely powerful for identifying non-coding regulatory regions located in intergenic or intronic regions. However, non-coding functional elements can also be located within coding region, as is common for exonic splicing enhancers, some transcription factor binding sites, and RNA secondary structure elements affecting mRNA stability, localization, or translation. Since these functional elements are located in regions that are themselves highly conserved because they are coding for a protein, they generally escaped detection by comparative genomics approaches.

**Results:**

We introduce a comparative genomics approach for detecting non-coding functional elements located within coding regions. Codon evolution is modeled as a mixture of codon substitution models, where each component of the mixture describes the evolution of codons under a specific type of coding selective pressure. We show how to compute the posterior distribution of the entropy and parsimony scores under this null model of codon evolution. The method is applied to a set of growth hormone 1 orthologous mRNA sequences and a known exonic splicing elements is detected. The analysis of a set of *CORTBP2 *orthologous genes reveals a region of several hundred base pairs under strong non-coding selective pressure whose function remains unknown.

**Conclusion:**

Non-coding functional elements, in particular those involved in post-transcriptional regulation, are likely to be much more prevalent than is currently known. With the numerous genome sequencing projects underway, comparative genomics approaches like that proposed here are likely to become increasingly powerful at detecting such elements.

## Background

Vertebrate genomes are now recognized as containing a huge number of non-coding functional regions, a large fraction of which is likely to be involved in regulating the various steps of gene expression [1-4]. While most of the attention has been centered on understanding the regulation of transcription, post-transcriptional regulatory mechanisms now appear to be more important than originally thought. Cis-regulation of pre-mRNA splicing is believed to be operated by splicing factors binding intronic and exonic splicing enhancers and helping to include or exclude specific exons from the transcript [5,6]. Post-splicing, parts of the mature mRNA often folds into some RNA secondary structure that determines the level of mRNA degradation [7] as well as mRNA localization [8]. Translational efficiency and accuracy have been shown to be largely determined by the choice of synonymous codon, thus imposing some selective pressure on the codons of certain genes [9]. Translation is also known to be affected by certain secondary structure elements in the mRNA [10]. While most of the known examples of formation of functional secondary structure are restricted to the 5' and 3' UTRs, the coding portion of the mRNA has also been shown to form functional structures [11]. Finally, there are also examples of transcription factor binding sites located in coding exons (e.g. in CD28 [12]). The method presented here should allow the detection of many of these functional elements, which we call coding regions under non-coding selection (CRUNCS). To this point, the computational methods that have proven the most valuable for identifying non-coding functional regions are based on comparative genomics. The guiding principle of this family of approaches is that functional features of a DNA sequence tend to evolve slower than non-functional ones, because of selective pressure. This simple idea is at the core of phylogenetic footprinting, a method that compares orthologous regulatory DNA regions to identify short conserved motifs likely to be transcription factor binding sites [13,14]. The key here is that most of the DNA in promoter regions is non-functional, with the exception of the regulatory elements we are interested in. The same reasoning applies to the detection of intronic splicing enhancers [15]. With the ongoing sequencing of a large number of vertebrate genomes [16], the power of these methods is quickly improving and, coupled with algorithmic improvements [17], they are now able to detect very short regions under selection, or regions under weak selection.

The search for CRUNCS is more challenging. Although the same "conservation implies function" principle applies in this case, it needs to be used more cautiously. Indeed, CRUNCS are *not *located in non-functional sequences as are, for example, most known transcription factors binding sites, but rather in *coding *regions. This means that the sequence conservation observed in exons may be the result of two types of selective pressures. The first one is the pressure to maintain the function of the protein encoded by the gene, which probably explains most of the sequence conservation observed in coding regions. The second type of selective pressure applies only to CRUNCS, which are required to maintain their regulatory role. To apply phylogenetic footprinting to the detection of CRUNCS, one needs to determine which type of selective pressure is responsible for the sequence conservation observed.

The method suggested here takes a conservative approach to the problem. Given a set of aligned orthologous coding sequences, we first evaluate the degree of conservation of each column of the alignment, using either a parsimony score or an entropy score. We then put the burden of explaining the conservation observed as much as possible on the shoulders of the selective pressure on the protein product. Because most amino acids are encoded by many synonymous codons, amino acid selective pressure leaves room for some sequence variation. A region of the sequence will be predicted to be an CRUNCS only if the conservation observed cannot be explained solely by the need for conservation of the encoded amino acids. The method introduced here build a mixture model of codon evolution, and then uses it as a null model to assess the significance of the observed degree of conservation. We illustrate our approach on two sets of orthologous vertebrate genes (growth hormone 1 and *CORTBP2*) and compare it to a related approach by Blanchette [18].

## Results and Discussion

Given a multiple alignment of orthologous mRNA sequences, our goal is to identify alignment columns that are conserved beyond what would be expected by chance if the corresponding sites were evolving only under the selective pressure on the amino acid they contribute to encode. Such sites are likely to be under non-coding selective pressure. This section, which constitutes the main contribution of this paper, is structured as follows. First, we define two commonly used sequence conservation scoring methods: the entropy, and the parsimony score. We then describe a methods for assigning a p-value to a given entropy or parsimony score, under null models of evolution of codons that are only under coding selective pressure. Under this method, we model codon evolution as a mixture of codon substitution models and use these models to assign a posterior p-value to a given conservation score.

### Two measures of sequence conservation

A number of methods have been proposed to measure the degree of conservation of a set of orthologous sequences and to identify regions under selective pressure (see [19] for an evaluation of some of these methods for finding regulatory elements in intergenic regions). In this paper, we consider two such methods, the entropy score and the parsimony score, and later show how to assess the statistical significance of these scores in the context of coding regions.

#### Entropy

In the area of transcription factor binding sites detection, a popular method for evaluating sequence conservation is the entropy (see, for example, [20]). Given a set of orthologous nucleotides *x*_1_, *x*_2_, ...,*x*_*n *_from *n *different species, the entropy measures the distance between the observed frequency of A, C, G, and T's at that site and the uniform distribution: *entropy *(*x*_1_, *x*_2_, ...,*x*_*n*_) = -∑*α*∈{*A*,*C*,*G*,*T*} *f*_*α *_log_2_(*f*_*α*_), where *f*_*α *_is the relative frequency of nucleotide *α*. Perfectly conserved sites have an entropy of zero, while the worst levels of conservation obtain a score of 2.

#### Parsimony score

**Figure 1:**
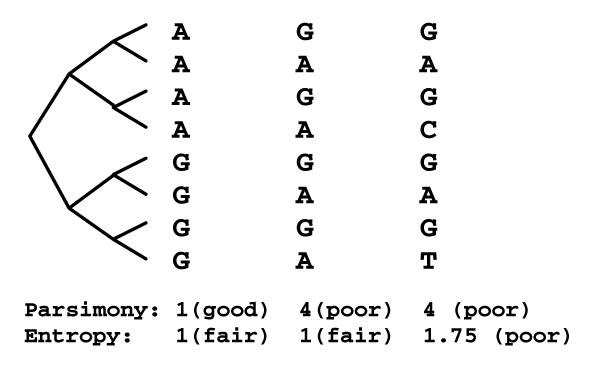
Example of alignment columns where parsimony score and relative entropy differ greatly in their assessment of sequence conservation.

A major drawback of the entropy score is that it does not take into consideration the phylogenetic relationships among the sequences being compared, and indeed the method is mostly used for motif discovery within a single species. An alternative to the entropy score is the parsimony score [21]. Given a set of orthologous nucleotides *x*_1_, *x*_2_, ..., *x*_*n *_and a phylogenetic tree *T *whose leaves are labeled with these nucleotides, the parsimony score is defined as the minimum number of substitutions, performed along the branches of the tree *T*, that can explain the set of nucleotides observed at the leaves. It is thus a lower bound on the actual number of substitutions at that site, and, in cases where this number is not too large compared to the number of branches in the tree, it is a fairly good estimate of the actual number of substitutions that occurred at that site. The parsimony score of a set of *n *nucleotides can be computed in time *O*(*n*) using Sankoff's algorithm [22] or Fitch's algorithm [21]. Figure 1 gives an example of orthologous nucleotides whose conservation is well characterized by either the entropy or the parsimony scores. Entropy and parsimony scores attempt to measure the total selective pressure on a given site. While, for non-coding regions, this selective pressure can be assumed to come completely from the presence of non-coding functional elements, this is not the case in coding regions. The rest of this paper describes how to measure the statistical significance of a certain conservation (entropy or parsimony) score when the site under study lies within a coding region.

### Conservation p-values under a mixture of codon models

In this section, we introduce a null model of coding sequence evolution that consists of a mixture of codon substitution models representing the evolution of codons that are under different types of *purely *coding selective pressure. We then show how to compute posterior p-values for the entropy and parsimony scores of orthologous sequences evolving under those models.

#### Mixture models for codon evolution

Different positions in a protein sequence are usually subject to different types of coding selective pressures. Some are constrained to have a specific amino acid (e.g. the active site in a zinc finger has to be a cystein), while others are free to have any residues with some particular chemical properties (say, a hydrophobic residue), and still others are under little or no selective pressure at all. Selective pressure on amino acids translates into selective pressure on codons, which explains part of the sequence conservation observed at the mRNA level in coding exons. We describe a mixture model of amino acid evolution, and the corresponding mixture model of codon evolution. We derive a set of 50 amino acid substitution rate matrices , ..., , together with codon rate matrices ,..., , describing the substitution rates for as many classes of selective pressures on amino acids. The choice of modeling codon evolution with only 50 classes is a compromise between an highly accurate modeling of codon evolution (which would most likely require a larger number of classes [23]) and constraints on computing time. Tests carried out with 100 classes instead of 50 resulted in very similar results (data not shown).

We learn amino acid functional categories using the Pfam database of amino acid sequence alignments of protein domains [24]. We start by learning the amino acid stationary distribution of each rate category. We then use these distributions to classify the Pfam alignment columns, and use this classification to estimate the amino acid and codon substitution rate matrices for each class.

**Figure 2:**
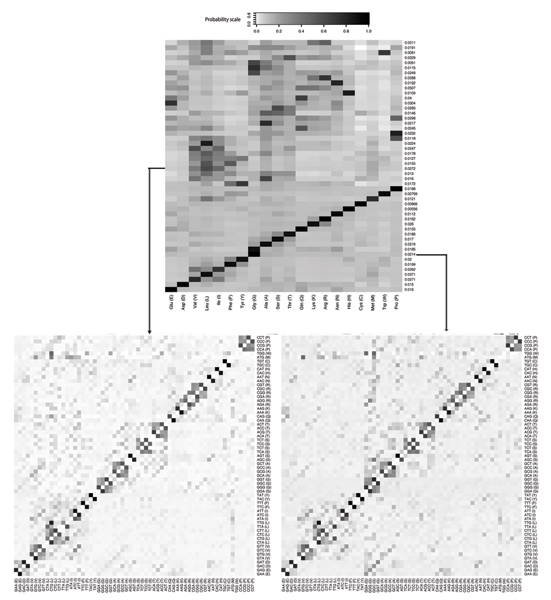
Mixture of codon substitution models based on amino acid functionality classes. (Top) Stationary distributions estimated from the Pfam database for the 50 functional classes. Each row corresponds to one class. The numbers on the right are the prior probability of each class. (Bottom) Two examples of codon rates matrices, where dark cells correspond to high substitution rates and light cells to low rates. The left matrix corresponds to a functional class that favors hydrophobic residues (ILV), while the right matrix comes from a class that favors the glycine amino acid (G).

The stationary amino acid distributions *π*_1_, *π*_2_, ..., *π*_50 _and class prior probability distribution *τ *are estimated using an unsupervised Expectation-Maximization algorithm (see Methods) in order to fit the Pfam alignments as closely as possible. Figure 2 (top) shows the amino acid distribution obtained for each of the 50 classes (see also Supplementary data). We observe that most of the expected functional classes are present among our 50 classes. First, for each amino acid, there is at least one category where that amino acid has a probability at least 0.5. These correspond to positions that tolerate little variation outside of that particular amino acid. Less constrained categories include several combinations of residues with similar properties: hydrophobic residues (ILV), small neutral residues (AST), aromatic residues (YF), positively charged residues (KR), etc. The class of negatively charged amino acids (DE) is surprisingly not directly represented, although these two amino acids show up together in several more weakly defined classes. Various categories correspond to positions under little selective pressure. Many of our classes are similar to those reported by Sjolander et al. [25] using a related procedure.

#### Amino acid and codon substitution rate matrices

For each of the 50 classes above, an amino acid substitution rate matrix and a codon substitution rate matrix are derived. We first compute the probability of each Pfam alignment column to belong to each of the classes, and use these to estimate the probability of amino acid and codon transitions between human and mouse sequences. Rate matrices are then derived from these empirically estimated transition probabilities matrices. The detailed procedure is described in Methods.

Figure 2 (bottom) shows two of the codon rate matrices obtained. Note how mutations toward codons encoding favored amino acids occur at a high rate, whereas substitutions away from those are rare. Notice that these rate matrices automatically take into account codon biases, as they are built from mRNA sequences. We make the assumption that the codon biases of human and mouse, which are reflected in our rate matrices, are representative of the codon biases in the other species used in our analysis. This assumption appears to hold quite well within mammals and birds, and to a lesser extent within vertebrates in general [26]. However, we recognize the fact that some genes (e.g. those requiring a high rate of translation) may have codon biases that are stronger than the average, resulting in an unexpected degree of conservation. Still, since this type of selective pressure would most likely apply to the entire transcript, it would be easily detectable using our approach, and would not result in false identification of other types of non-coding elements.

#### Distribution of conservation scores

We now return to the problem of identifying regions under non-coding selective pressure in a multiple alignment of orthologous coding mRNA sequences  = *X*_1 _... *X*_*m*_, where *X*_*i *_is the triplet of alignment columns corresponding to the *i*-th codon in the alignment. Let *X*_*i*_(*j*) be the codon observed in species *j *∈ {1...*s*}, where *s *is the number of column triplets in the alignment, and let *X*_*i*,*p*_(*j*) be the nucleotide at position *p *(*p *= 1, 2 or 3) in that codon. Given a column of orthologous codons *X*_*i*_, we want to assess whether the conservation observed at position *p *of the codon is unexpected. To this end, we compute the posterior p-value of the observed conservation score (entropy or parsimony score) of that codon position, under the null hypothesis that the columns are only under coding selective pressure.

To describe more formally our null model of sequence evolution, we need to introduce some notation. Let *T *= (*V, E*) be a binary phylogenetic tree with vertices *V*, edges *E*, root *r*, and with leaves numbered 1,2,...,*n*. Let *λ*(*u*, *v*) be the length of the branch going from node *u *to node *v*, let *a*(*c*) be the amino acid encoded by codon *c*, and let **Q **be some codon substitution rate matrix. The codon transition probability matrix for a branch (*u*,*v*) is given by **P**_(*u*,*v*) _= *e*^*λ*(*u*,*v*)^^**Q **^[27]. Let *b*(c) be the background probability of codon *c*, which is assumed to be the stationary distribution of **Q**. These three parameters (*T*, *λ*, **Q**) describe a process that generates random but related codons at the leaves of the tree *T*, by drawing a codon from the stationary distribution of **Q **at the root of *T *and letting it mutate along the branches of the tree using the appropriate transition probability matrices. Let *C*(*u*) be the random variable representing the codon that has been generated by this process at node *u *of the tree.

We are interested in computing the distribution of the conservation score of a given position *p *of a set of random orthologous codons generated at the leaves of the tree. We start by showing how to compute this distribution for the entropy score *entropy*(*C*_*p*_(1),*C*_*p*_(2), ..., *C*_*p*_(*n*)), and later show the modifications required to do the same for the parsimony score. For a fixed codon position *p *∈ {1,2,3}, and for any node *u *∈ *V*, let *Y*_*u *_= (*Y*_*u*_(A), *Y*_*u*_(C), *Y*_*u*_(G), *Y*_*u*_(T)) be a random multivariable where *Y*_*u*_(*α*) is the number of nucleotides of type *a *at position *p *of the codons at the leaves of the subtree rooted at *u*. Notice that *Y*_*u *_is only a function of the codons at the leaves of subtree(*u*), and not of those at the internal nodes of subtree(*u*). The p-value of a certain entropy score *e *for position *p *is obtained by summing the probabilities of all values of *Y*_*r *_that yield an entropy score *e *or better:



We will show how to compute Pr[*Y*_*u *_= (*y*_*a*_, *y*_*c*_, *y*_*g*_, *y*_*t*_)|*C*(*u*) = *k*], for every node *u *and all choices of *y*_*a*_, *y*_*c*_, *y*_*g*_, *y*_*t *_and *k*, using a dynamic programming algorithm visiting the nodes of *T *in post-order. When *u *is a leaf, we have



Define (*y*_*a*_, *y*_*c*_, *y*_*g*_, *y*_*t*_) ⊕ (*z*_*a*_, *z*_*c*_, *z*_*g*_, *z*_*t*_) : = (*y*_*a*_*+ z*_*a*_, *y*_*c*_*+ z*_*c*_, *y*_*g*_*+ z*_*g*_, *y*_*t*_*+ z*_*t*_). Now, let *u *be an internal node with children *v *and *w*. Notice that *Y*_*u *_= *Y*_*v *_⊕ *Y*_*w*_. We compute the desired conditional probabilities at node *u *based on those at nodes *v *and *w*:



Implementation optimizations and computational complexity analysis are given in Methods.

#### Distribution of parsimony scores

The method described in the previous section can be surprisingly easily modified to compute the conditional p-value of parsimony scores instead of that of the entropy score. We need to redefine the random variable *Y*_*u *_= (*y*_*a*_, *y*_*c*_*, y*_*g*_*, y*_*t*_) so that *y*_*α *_is now the parsimony score obtained for the nucleotides at position *p *of codons at the leaves of the subtree rooted at *u*, assuming that the nucleotide at the ancestral node *u *is required to be *a*. Note that this set of four numbers per node is exactly that computed by the Sankoff algorithm for computing parsimony scores [22]. We also redefine the ⊕ operator as

(*y*_*a*_, *y*_*c*_, *y*_*g*_, *y*_*t*_) ⊕ (*z*_*a*_, *z*_*c*_, *z*_*g*_, *z*_*t*_) = (min(*y*_*a *_+ *z*_*a*_, *y*_*a *_+  + 1,  + *z*_*a *_+ 1, +  + 2),

min(*y*_*c *_+ *z*_*c*_, *y*_*c *_+  + 1,  + *z*_*c *_+ 1,  +  + 2),

min(*y*_*g *_+ *z*_*g*_, *y*_*g *_+  + 1,  + *z*_*g *_+ 1,  +  + 2),

min(*y*_*t *_+ *z*_*t*_, *y*_*t *_+  + 1,  + *z*_*t *_+ 1,  +  + 2))

where  = *min*_*j*≠*i*_*y*_*j*_. Notice that this is again in direct analogy to Sankoff's algorithm. Again, *Y*_*u *_= *Y*_*v *_⊕ *Y*_*w *_and we get *parsimony *(*C*_*p*_(1), (*C*_*p*_,(2),..., *C*_*p*_(*n*)) = *min*(*Y*_*r*_). With these redefinitions, Equation 2 holds without any modifications needed. We get



### Posterior distributions of conservation scores

Having shown how to compute the p-value of a given entropy or parsimony score under a fixed codon rate matrix, it is simple to compute posterior p-values for the case where the functional class is not known in advance. Consider a given set of aligned codons *X*_*i *_= (*X*_*i*_(1), ..., *X*_*i*_(*n*)) encoding the set of amino acids *A*_*i *_= (*A*_*i*_(1), ..., *A*_*i*_(*n*)). Define the unobserved variables *Z*_*i*,*k *_= 1 if the site *i *belongs to functional class *k*, and zero otherwise. We first compute the posterior probability of each *Z*_*i*,*k*_, given the observed amino acids at site *i*:



where Pr[*A*_*i*_(1), ..., *A*_*i*_(*n*)|*Z*_*i*,*k *_= 1, ] is computed using Felsenstein's algorithm [28], with rate matrix . Finally, we obtain the posterior estimate *pv*_*post*_(*i*,*p*) for the p-value of the entropy score *e *observed at position *p *of codon *i*, by summing over all classes:



and similarly for parsimony scores p-values.

### Conditional p-values

**Figure 3:**
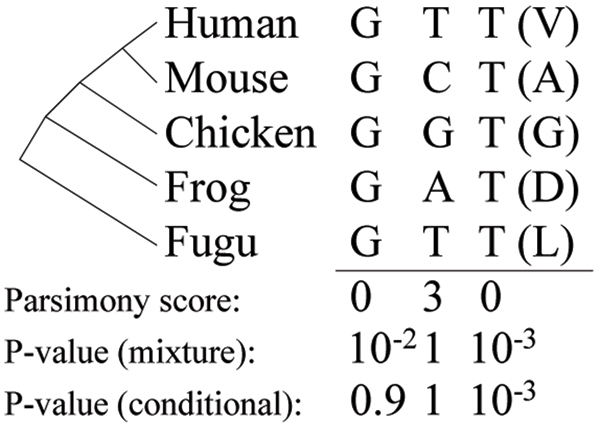
Example of codons where the posterior p-values under the mixture of codon models differs significantly from p-values obtained from the conditional p-value method. Amino acids V,A,G,D,L do not have any common properties, so, under the codon mixture model, the column is assigned to a functional class with little selective pressure, explaining why the p-value produced for the first codon position is small. In contrast, under the conditional p-value approach, the amino acids almost completely determine the first codon position (the only exception being the Fugu codon, encoding a leucine, which could have used a T at the first position), so a poor p-value is reported.

An alternative to trying to guess the type of selective pressure under which a given codon evolves is to use a single codon rate matrix but subject to the constraint that the random codon generated at each leaf has to encode the amino acid that was actually observed at that leaf. This approach was originally proposed by Blanchette [18]. This model does not rely on amino acid classifications and in fact allows sites to change function during their evolution. By conditioning on the observed amino acids at the leaves of the tree, we ask: given that in species *j*, the codon *had *to encode amino acid *a*(*X*_*j*_(*i*)), for each leaf *j *∈ {1, ..., *n*}, is the conservation observed in *X*_*i *_surprising? Notice how, compared to the mixture model approach, this model transfers the responsibility of sequence conservation even more onto the shoulders of coding selection. See Figure 3 for an example. Mathematical details are provided in Methods.

#### A sliding window approach

Until now, we have shown two ways to compute p-values for individual alignment columns. Since most non-coding functional elements are expected to span several consecutive positions (5-15nt for transcription factor binding sites and exonic splicing enhancers, and up to a few hundred nucleotides for RNA secondary structure elements), we can improve the sensitivity of the method by using a simple sliding window approach. For each position *i*, let *pv*(*i*) be the p-value obtained for the *i*-th column of the alignment (not the *i*-th codon), and let *w *be the width of the sliding window (assume *w *is odd, for simplicity). We compute *S*_*i *_= ∏_*j *= *i*- ⌊*w*/2⌋...*i *+ ⌊*w*/2⌋ _*pv*(*j*). If we assume that, under the null model, *pv*(*j*) is approximately uniformly distributed, a compounded p-value can be assigned to *S*_*i*_: *cpv*(*i*) = Pr[Product of *w *i.i.d. uniform (0,l) ≤ *S*_*i*_] = *S*_*i*_(1 + ∑_*j *= 1...*w*-1 _- ln(*S*_*i*_)^*j*^*/j*!). This is the type of p-value being reported in the Applications section. It should however be noted that although the uniformity assumption holds quite well for the third codon positions, the first two codon positions are often completely determined by the codon they encode, so the range of possible p-values they can take is quite small. This results in the compounded p-values being quite conservative.

#### Implementation

The algorithms were implemented in C++ and the program is available upon request. A number of optimizations described in [18] have also been implemented and make the program relatively fast. In particular, a caching mechanism allows to re-use the results of computations done on previous columns. This allows the program to handle very long sequences quickly. All analyses reported here have been obtained in less two hours of computation on a desktop machine.

### Analysis of simulated data

**Figure 4:**
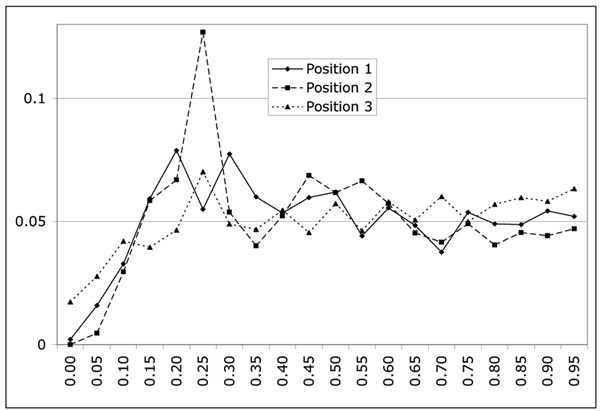
Distribution of the p-values obtained on a set of 50-kb sequences evolving according to the null model. The tree used for the simulation is the same as that for the GH1 dataset (see below).

We first verify that the p-values computed by our approach have the basic properties we would expect of them. To start, we confirm that sequences evolving under the null model obtain p-values that are approximately uniformly distributed. This would be a trivial statement if the functional category of each site was known, but in the absence of such prior knowledge, the uniformity of p-values under the null model is less obvious. To this end, we simulated the evolution of a 50 kb region of DNA, with each codon belonging to one of the 50 rate categories described above. Sequences were evolved along the branches of the 69-leaf phylogenetic tree derived from the GH1 data set described below. Figure 4 shows the distribution of posterior p-values obtained at each of the three codon positions. As desired, the distribution is nearly uniform. However, we observe a depletion of columns with low p-values (<0.1), in particular for codon positions 1 and 2. This is due to the fact that, at these positions, a column that is perfectly conserved is not particularly surprising, and obtains a p-value around 0.2–0.3. To obtain a p-value distribution that is closer to uniform, one would need to use a tree with much larger total branch length. Second, we study the power of our method to detect selection on sets of aligned codons that are perfectly conserved. As expected, the power of our method to detect CRUNCS depend on the amino acid encoded by the codon. Perfectly conserved codons encoding amino acid W cannot be detected by our approach. On the other hand, codon conservation is easiest to detect among four-fold and six-fold degenerate amino acids. Among those, codons encoding amino acids P and G, both of which have chemical properties making them more difficult to exchange for other amino acids, obtain higher p-values, because their conservation can be explained to a larger extent by the amino acid encoded.

#### Analysis biological data

We illustrate our approach on two sets of orthologous mRNA sequences: a set of 69 vertebrate growth hormone 1 (*GH1*) sequences, and a set of 13 vertebrate *CORTBP2 *(also known as *CTTNBP2*) sequences (see Supplementary data).

**Figure 5:**
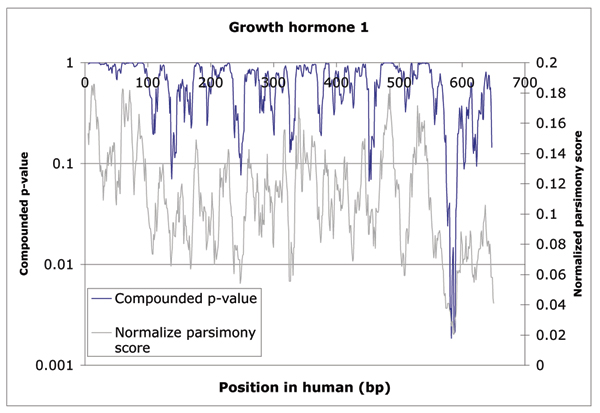
Dark curve: compounded parsimony p-values for the *GH1 *gene under the mixture model, using a window size of *w *= 9 nucleotides. Light curve: average parsimony score per alignment column, normalized by the number of leaves in the tree (which varies from site to site, due to gaps in the alignment).

*GH1 *was one of the first gene shown to harbor an exonic splicing enhancer, in cow [29]. The availability of a large number of orthologous sequences makes it ideal for our study. Figure 5 shows the compounded p-values obtained from the mixture-based method, for the parsimony score, using a sliding window with *w *= 9. The human region orthologous to a known exonic splicing enhancer in the cow, located around position 577 [29], is clearly identified, obtaining a compounded p-value of about 2 × 10^-3^. Although this region could have been identified on the basis of parsimony scores alone (gray curve on Figure 5), it is highlighted much more clearly by the posterior p-values.

**Figure 6:**
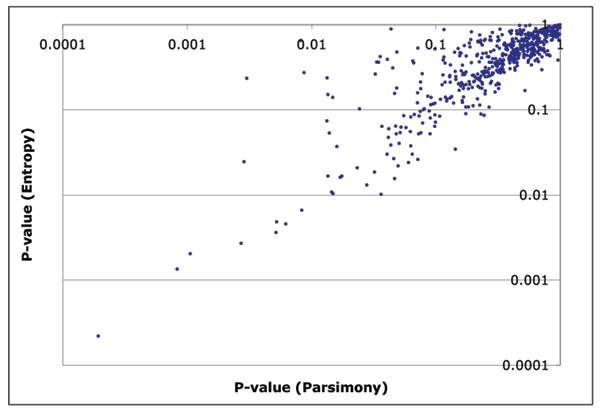
Comparison of the posterior p-values obtained from the entropy and parsimony scores, under the mixture of codon models, on the *GH1 *gene.

Figure 6 compares the posterior p-values obtained for the entropy and parsimony scores, under the mixture model of codon evolution, on the growth-hormone 1 data set. The correlation between the two is quite obvious, especially for very small p-values. This is in part due to the fact that alignment columns that are perfectly conserved obtain the same p-values from the two methods. On the other hand, many more sites obtain p-values between 0.01 and 0.1 using the parsimony score than using the entropy score. These are sites that have undergone a small number of substitutions early in vertebrate evolution, and that obtain a good parsimony score but a poor entropy score, as in Figure 1. In this data set, there are no sites that obtain a good entropy p-value and a poor parsimony p-value. Since the parsimony score p-values can be computed much faster and since they appear to be strictly more sensitive than entropy p-values, they seem to be the method of choice in most situations.

**Figure 7:**
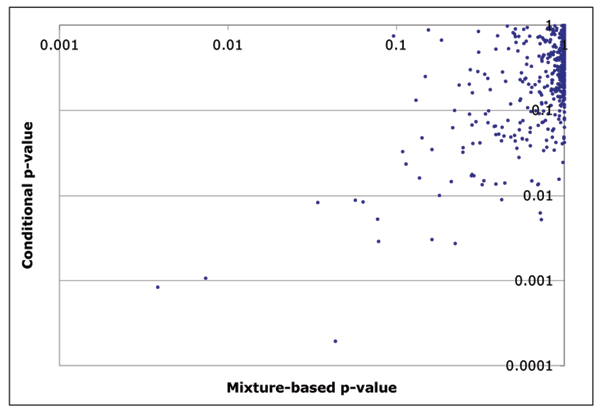
Comparison of the p-values obtained from the parsimony scores, under the mixture of codon models and under the conditional probability computation.

Figure 7 compares the p-values obtained for the parsimony score under the mixture model and to those obtained under the conditional p-value approach. Although the two are clearly correlated, there are some important differences. First, most of the p-values obtained for the 1st and 2nd codon positions under the conditional probability model are very close to 1, because these two codon positions are often completely determined by the amino acids observed at the leaves. Under the mixture model, the p-values obtained at these positions will depend on the variability of the amino acids observed, and in general will be smaller. This is also true, to a lesser extent, for the third codon position.

**Figure 8:**
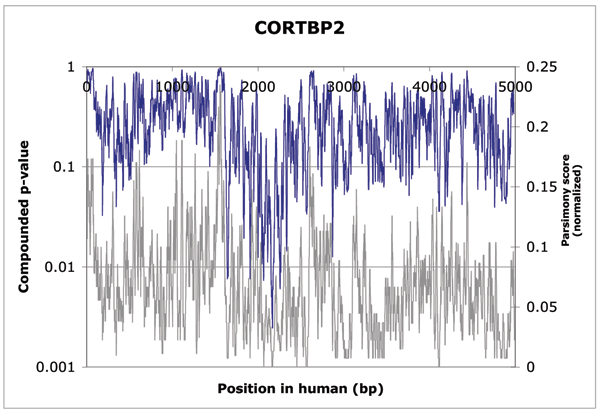
Dark curve: compounded parsimony p-values for the *CORTBP2 *gene, using a window size of *w *= 15 nucleotides. Light curve: average parsimony score per alignment column, normalized by the number of leaves in the tree (which varies from site to sites, due to gaps in the alignment).

Finally, the analysis of the *CORTBP2 *transcript is particularly intriguing. Little is known about the post-transcriptional regulation of this gene. We find that its mRNA contains a very large region (roughly between positions 1500 and 2200, see Figure 8 and Supplementary material), which obtain fairly low p-values. This region is much too large to be a binding site, and we conjecture that it may form some large RNA secondary structure affecting the pre-mRNA splicing or the mRNA stability, localization or translation. Notice that in this case, a simple parsimony score is not sufficient for identifying the region, as many other regions of the transcript are well equally well conserved.

## Conclusion

With the many genome sequencing projects rapidly producing vertebrate genomic data, comparative genomic approaches are becoming increasingly powerful. In the case of CRUNCS, additional data is often available in the form of ESTs and cDNAs. We believe that within one year or two, there will be sufficient data for accurate detection of CRUNCS in vertebrate genes, using methods like those described here. Once many CRUNCS will have been detected, the next step will of course be to assign functions to these elements. Although the last word will remain with experimentalists, we have good hopes that more advanced bioinformatics approaches will yield insights into these questions.

Finally, we expect that organisms that are under severe genome size constraints, in particular bacteria and viruses, will more often use CRUNCS. We believe that our approaches will prove particularly fruitful to analyze these genomes.

## Methods

### Estimating amino acid stationary distributions for each class

The Pfam database consists of a set of multiple alignments of homologous protein domain sequences. For some domains, the database contains several sequences that are very closely related. To reduce biases due to this over-representation, one member of any pair of domain sequences that share more than 60% identity is discarded. Let *D*_1_, ..., *D*_*m *_be the set of alignment columns in this reduced Pfam database, let *S*_*i *_be the number of species in alignment column *i*, let *D*_*i*_(*j*) be the amino acid from species *j *in column *i*, and let *E*_*i*_(*j*) be the codon encoding that amino acid in the mRNA of species *j*. Starting from Pfam 17.0, we have *m *= 2292182 amino acid alignment columns.

For simplicity, we assume that the amino acids in a given column are drawn independently from the same unknown amino acid distribution. Let *π*_1_, *π*_2_, ..., *π*_50 _be a set of amino acid distributions, where *π*_*k*_(*α*) is the probability of amino acid *a *in class *k*. Let *τ*(*k*) be the prior probability of class *k*. The class membership of column *i *is unknown. Let *Z*_*i*_(*k*) be a hidden variable that takes value 1 if column *i *belongs to class *k*, and 0 otherwise. We have Pr[*D*_*i*_|*Z*_*i*_(*k*) = 1] = .

We search for the distributions *π*_1_, ..., *π*_50 _and prior probabilities *τ *that maximize Pr[*D*_1 _... *D*_*m*_] = ∏_*i *= 1...*m *_Pr[*D*_*i*_] = ∏_*i *= 1...*m *_∑_*k *= 1..50 _Pr[*D*_*i*_|(*k*) = l]*τ*(*k*). This is achieved using a simple EM algorithm for learning mixtures of multinomial distributions, using the following update formulas to go from iteration *t *to iteration *t *+ 1.



and



where *N*_*i*_(*α*) is the number of occurrences of amino acid *α *in column *D*_*i *_and where *ζ *and *ζ*' are normalizing constants that ensure that the probabilities sum to one.

Initialized with noisy uniform distributions, the algorithm converges quickly (less than 50 iterations) to the local optimum depicted in Figure 2. Ten restarts from other random initial conditions converged to very similar distributions, so the first one was retained.

### Estimating amino acid and codon rate matrices

Once the amino acid distributions *π*_1_, ..., *π*_50 _and prior distribution *τ *are learned, we can use them to compute the posterior probability of each class *k *for each alignment column *D*_*i*_:



where *ζ*'' is another normalizing constant. For simplicity, the amino acid and codon substitution rate matrices  and  are estimated for each class *k *based only on the observed substitutions between human and mouse sequences (however, the class posterior probabilities of each column are based on all available species). Any column that does not include an entry for these two species is excluded. Let *E*_*i*_(*human*) and *E*_*i*_(*mouse*) be the codons encoding amino acid *D*_*i *_(*human*) and *D*_*i*_(*mouse*) in the corresponding mRNA sequences. The transition matrices are then estimated as follows:





Finally, we estimate the instantaneous rate matrix (*α*, *β*) = ln (*α*, *β*)/*t*_(*h*,*m*)_, where *t*_(*h*,*m*) _is the expected number of substitutions per site between human and mouse, in neutrally evolving DNA. Codon rate matrices are obtained in a similar manner.

Note that the codon substitution rates we obtain are slightly underestimating the true rates for sites evolving only under coding selective pressure, because some sequences in Pfam are likely to be evolving slower due to non-coding selective pressure. However, we expect that this underestimation is negligible as the fraction of such sites is likely to be small. In any case, underestimating the rates will only cause conservative estimates of the conservation p-values.

### Computing entropy and parsimony score p-values

The probabilities Pr[*Y*_*u *_= (*y*_*a*_, *y*_*c*_, *y*_*g*_, *y*_*t*_)|*C*(*u*) = *k*_*u*_] are stored in a hash table associated to each node, indexed by the quintuplet (*y*_*a*_, *y*_*c*_, *y*_*g*_, *y*_*t*_, *k*_*u*_). Only non-zero probabilities are stored. To compute these probabilities for a node *u *with children *v *and *w*, it is simpler to enumerate all pairs (*y*_*a*_, *y*_*c*_, *y*_*g*_, *y*_*t*_, *k*_*v*_), (*z*_*a*_, *z*_*c*_, *z*_*g*_, *z*_*t*_, *k*_*w*_) of quintuplets from the hash tables of the two children, and add the proper quantity to the entry ((*y*_*a*_, *y*_*c*_, *y*_*g*_, *y*_*t*_) ⊕ (*z*_*a*_, *z*_*c*_, *z*_*g*_, *z*_*t*_), *k*_*u*_) of the hash table at *u*, for all choices of *k*_*u*_. Please see [18] for more details.

To study the complexity of the resulting algorithm for the entropy p-value computation, observe that the hash table associated with node *v *whose subtree contains *l*(*v*) leaves will contain at most  ∈ *O*(*l*(*v*)^3^) non-zero entries and thus computing all entries of the hash table for node *u *takes *O*(*l*(*v*)^3^*l*(*w*)^3^) time. Depending on the topology of *T*, computing the hash tables of all nodes takes between *O*(*n*^4^) time for a completely skewed tree and *O*(*n*^6^) time for a balanced tree. The time complexity analysis applies to both the posterior p-value and the conditional p-value computations. However, for posterior p-values, this computation needs to be repeated 50 times, once for each rate matrix.

The hash table implementation works well in the case of parsimony score p-value computation too, especially with the following optimizations. First, for binary trees, the only choices of *y*_*a*_, *y*_*c*_, *y*_*g*_, *y*_*t *_that may have non-zero probability are those where |*y*_*i *_– *y*_*j*_| ≤ 2 for all *i, j *∈ {A, C, G, T} (this is a direct consequence of the ⊕ operator). When evaluating the p-value for a parsimony score *ψ*, choices of *y*_*a*_, *y*_*c*_, *y*_*g*_, *y*_*t *_such that *min*(*y*_*a*_, *y*_*c*_, *y*_*g*_, *y*_*t*_) > *ψ *are not affecting the final p-value and can be safely ignored. Therefore, the hash table associated with node *v *contains *O*(*ψ*) = *O*(*n*) entries, and computing all entries for node *u *from those of nodes *v *and *w *take *O*(*l*(*u*) · *l*(*v*)). Thus, one can compute all entries of all tables in *O*(*n*^2^), irrespective of the tree topology. More details can be found in [18].

### Conditional P-values

The method of conditional p-values, introduced by Blanchette [18], can be summarized as follows. The method computes the following conditional p-value:

*pv*_*cond*_(*i, p*) = Pr[*entropy*(*C*_*p*_(1), ...,*C*_*p*_(*n*)) ≤ *entropy*(*X*_*i*_,_*p*_(1), ..., *X*_*i*_,_*p*_(*n*))*|a*(*C*(1)) = *a*(*X*_*i*_(l)), ..., *a*(*C*(*n*)) = *a*(*X*_*i*_(*n*))]

This p-value can be computed in a manner that is similar to the Equation 2. Let us denote by *l*(*u*) the set of leaves in the subtree rooted at *u *and let us write the set of conditions for the subtree rooted at *u *as *A*(*u*) = ∧_*j*∈*l*(*u*)_(*a*(*C*(*j*)) = *a*(*X*_*i*_(*j*))). We get



and



where Pr[*C*(*u*) = *k|A*(*u*)], is computed using Felsenstein's algorithm and Bayes rule. As before, a dynamic programming algorithm proceeding in a post-order traversal of the tree allows the computation of all terms required.

## Authors' contributions

Chen implemented the program for the posterior p-value computation, for learning amino acid functional classes, and for learning the rate matrices associated to each class, and did some of the data analysis. Blanchette was responsible for the original ideas and mathematical derivations, for some of the data analysis, and for writing the paper.

## Supplementary data

Multiple alignments, phylogenetic trees and detailed results are available at [30].
